# Assessing immune phenotypes using simple proxy measures: promise and limitations

**DOI:** 10.1093/discim/kyae010

**Published:** 2024-06-28

**Authors:** Alexander E Downie, Ramya S Barre, Annie Robinson, Jennie Yang, Ying-Han Chen, Jian-Da Lin, Oyebola Oyesola, Frank Yeung, Ken Cadwell, P’ng Loke, Andrea L Graham

**Affiliations:** Department of Ecology and Evolutionary Biology, Princeton University, Princeton, NJ, USA; Department of Ecology and Evolutionary Biology, Princeton University, Princeton, NJ, USA; Department of Microbiology, Immunology, and Molecular Genetics, University of Texas Health Sciences Center at San Antonio; San Antonio, TX, USA; Department of Ecology and Evolutionary Biology, Princeton University, Princeton, NJ, USA; Department of Ecology and Evolutionary Biology, Princeton University, Princeton, NJ, USA; Kimmel Center for Biology and Medicine at the Skirball Institute, New York University Grossman School of Medicine; New York, NY, USA; Department of Microbiology, New York University Grossman School of Medicine; New York, NY, USA; Institute of Biomedical Sciences, Academia Sinica, Taipei City, Taiwan; Department of Biochemical Science and Technology, College of Life Science, National Taiwan University, Taipei City, Taiwan; Center for Computational and Systems Biology, National Taiwan University, Taipei City, Taiwan; Laboratory of Parasitic Diseases, National Institute for Allergy and Infectious Diseases, National Institutes of Health; Bethesda, MD, USA; Kimmel Center for Biology and Medicine at the Skirball Institute, New York University Grossman School of Medicine; New York, NY, USA; Kimmel Center for Biology and Medicine at the Skirball Institute, New York University Grossman School of Medicine; New York, NY, USA; Department of Microbiology, New York University Grossman School of Medicine; New York, NY, USA; Division of Gastroenterology and Hepatology, Department of Medicine, University of Pennsylvania Perelman School of Medicine, Philadelphia, PA, USA; Department of Systems Pharmacology and Translational Therapeutics, University of Pennsylvania Perelman School of Medicine, Philadelphia, PA, USA; Department of Pathology and Laboratory Medicine, University of Pennsylvania Perelman School of Medicine, Philadelphia, PA, USA; Kimmel Center for Biology and Medicine at the Skirball Institute, New York University Grossman School of Medicine; New York, NY, USA; Department of Microbiology, New York University Grossman School of Medicine; New York, NY, USA; Laboratory of Parasitic Diseases, National Institute for Allergy and Infectious Diseases, National Institutes of Health; Bethesda, MD, USA; Department of Ecology and Evolutionary Biology, Princeton University, Princeton, NJ, USA; Santa Fe Institute; Santa Fe, NM, USA

**Keywords:** ecoimmunology, antibodies, lymphocytes, disease ecology

## Abstract

The study of immune phenotypes in wild animals is beset by numerous methodological challenges, with assessment of detailed aspects of phenotype difficult to impossible. This constrains the ability of disease ecologists and ecoimmunologists to describe immune variation and evaluate hypotheses explaining said variation. The development of simple approaches that allow characterization of immune variation across many populations and species would be a significant advance. Here we explore whether serum protein concentrations and coarse-grained white blood cell profiles, immune quantities that can easily be assayed in many species, can predict, and therefore serve as proxies for, lymphocyte composition properties. We do this in rewilded laboratory mice, which combine the benefits of immune phenotyping of lab mice with the natural context and immune variation found in the wild. We find that easily assayed immune quantities are largely ineffective as predictors of lymphocyte composition, either on their own or with other covariates. Immunoglobulin G (IgG) concentration and neutrophil-lymphocyte ratio show the most promise as indicators of other immune traits, but their explanatory power is limited. Our results prescribe caution in inferring immune phenotypes beyond what is directly measured, but they do also highlight some potential paths forward for the development of proxy measures employable by ecoimmunologists.

## Graphical Abstract

**Graphical Abstract:**
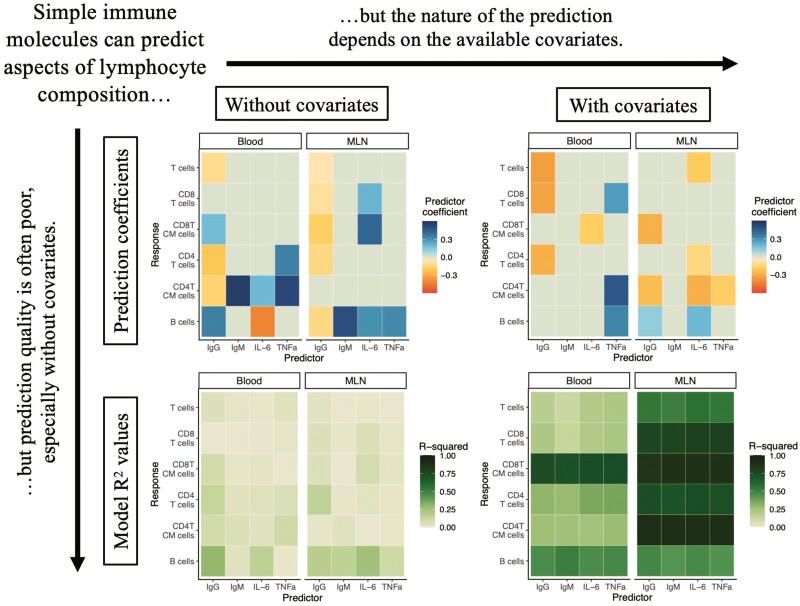

## Introduction

A topic of increasing interest in ecology and evolution is the description of immune system variation in nature and the consideration of the ecological and evolutionary forces shaping said variation [1, 2]. There is variation in many disparate parts of the immune system: immune cell composition [3–6], antibody persistence [7, 8], immune response magnitude [9–12], and gene and protein expression profiles in response to antigenic challenges [3, 13, 14]. Researchers have also described a wide variety of genetic variants affecting immune defenses [15], such as in antigen recognition proteins like the major histocompatibility complex (MHC) [16, 17], antibodies [18], and toll-like receptors (TLRs) [19]. Immune variation can take even more dramatic forms. Some taxa, such as members of the fish order Gadiformes (cod and relatives) and the deepsea ceratioid anglerfish, have lost or pseudogenized the genes coding for certain antigen recognition proteins [20, 21], and the evolution of male pregnancy in Syngnathiformes (pipefish and relatives) is associated with the elimination or pseudogenization of important genes associated with adaptive immune defenses, like *CD4* and *CD8b* [22]. Naked mole-rats (*Heterocephalus glaber*) may lack natural killer cells [23]. Other cell types are unique to certain taxa, like an unusual lineage of T cells so far only identified in marsupials [24]. The question of how evolution has produced and maintained this variation is an important one for immunologists, disease ecologists, and evolutionary ecologists.

Many correlates and drivers of such variation have been proposed. For example, life history is commonly put forward as a key covariate of immune defense, with particular immune strategies postulated to be associated with different life histories or ecologies [25–29]. Testing such theories requires an accurate description of immune phenotypes in natural populations. Many methods can be used to characterize the immune phenotypes of wild animals (see [30] for a comprehensive review). These methods vary in the type and amount of sample and the equipment and sampling conditions required. Many classic laboratory immunological techniques are simply not feasible with wild animals: the amount of blood or type of tissue required may not be possible to take without killing the animal, the sampling conditions may not allow for the correct preparation and preservation of samples, and/or the reagents required for a particular technique—flow cytometry, for example—may not exist for that species. Thus, although great immunological detail can be achieved in certain systems and ideal sampling conditions, researchers in wild animals or non-laboratory settings often have only a limited palette of techniques, like antibody assays and white blood cell counts, that can be used for any species [30, 31]. Such assays directly measure only a few quantities, like the total concentration of IgG in the bloodstream [1, 32, 33], or the concentration of antibodies of one isotype against a particular target [34, 35], or the relative abundance of the five most common leukocyte types routinely quantified in white blood cell differential counts: lymphocytes, neutrophils, basophils, eosinophils, and monocytes [36, 37]. These measures we refer to as “widely-employable” because they can be performed relatively easily in samples collected from non-model organisms or in wild, settings—for example, neutrophil-lymphocyte ratio (NLR) has been measured for a wide variety of mammal species [36]. Such quantities can certainly prove informative for eco-immunological or immunoepidemiological studies. But most other quantities (e.g. the frequencies of CD8+ memory T cells or their memory states) are inaccessible via direct measurement in wild animals, though many immunologists would argue that such data are extremely important for characterizing immune phenotypes.

Yet it is possible that some of the widely employable assays could enable researchers to infer other aspects of immune phenotype that cannot be measured in most systems and/or settings. Can traits that are more easily measured be effective proxies for other immune traits and even, perhaps, overall immune state? For example, do antibody concentrations correlate with immune memory state or overall investment in immune defenses, as some have hypothesized [26, 28]? Lymphocytes play key roles in directing immune responses, constitute immune memory, and are a particularly large energetic drain [26], and are therefore of strong interest to ecoimmunologists. They are traditionally characterized in humans and laboratory mice using flow cytometry, which relies on identifying cell surface markers, but flow cytometry reagents are generally only available for model organisms, such as humans, mice, or rhesus macaques, and these reagents typically do not cross-react well for use in other species, and conditions for flow cytometry preparation may also not be available when studying wild animals [30]. The identification of proxies for lymphocyte phenotypes and other aspects of immune defense would greatly help researchers to understand better the implications and limitations of the immunological data they gather from wild animals.

Here we assess whether a set of widely employable immune measures can serve as proxies for other aspects of immune phenotype. We examined this question using data drawn from rewilded laboratory mice, which are laboratory mice that have been raised in standard laboratory conditions before being released into outdoor enclosures with natural vegetation, weather, and dietary variation. Rewilded mice combine the control and access of a model organism, having documented exposure histories, well-mapped genetics amenable to manipulation, and an extensive capacity for phenotyping, with the chaos and ecologically-relevant conditions of a natural setting, making them an excellent venue for studying immune defenses [38–40]. Rewilding, in particular, provides immune systems with some of the microbial stimulation and other environmental experiences of wild animals, while also permitting variation in factors like social interaction that help structure immune variation [41]. Drawing on datasets from multiple experiments that use a mixture of mouse strains, different durations of rewilding, population densities, etc., we tested whether widely employable ecoimmunology measures—here, serum antibody and cytokine concentrations, as well as white blood cell differentials—can predict aspects of lymphocyte populations in the blood and mesenteric lymph nodes (MLNs) (drawing upon detailed immunological data first reported in [42–45]). We then consider the implications of the correlations, and lack thereof, for researchers studying immune defenses in the wild.

## Materials and methods

We drew our data for these analyses from rewilded laboratory mice used in multiple experiments investigating how rewilding alters mouse immune phenotypes. These experiments took place in the summer of 2017 (described in [43, 45]), the summer of 2019 (described in [42]), and the summer of 2021 (described in [41, 44]). Data from a total of 193 mice across these three experiments are included here. Our lymphocyte measures were previously published in [41–45], as were the serum cytokine measures and the neutrophil–lymphocyte ratio (NLR) measures for the 2021 experiment; the serum antibody concentrations for all mice and NLR for mice from the 2019 experiment are newly reported here. Each experiment used the same basic structure, with some variation depending on the precise goals of that year’s experiment.

### Rewilding procedures

Laboratory mice were born and raised in standard lab-husbandry conditions to 4–6 weeks of age before being released into outdoor enclosures, each ~180 m^2^. These enclosures, described in [46], are triangular in shape and surrounded by walls 1.5 m in height and extending 80 cm below the surface. Exterior walls are topped with an electric fence to keep out terrestrial predators, and aerial predators are excluded by means of fishing lines strung above each enclosure with sections of aluminum pie plates attached, to dazzle and prevent entry by flight. Camera trap footage and mouse RFID activity records suggest that these measures have been consistently effective for predator exclusion. Each enclosure contains a small wooden hut (180 cm × 140 cm × 70 cm); vegetation is allowed to grow freely except for a strip along the inside of the enclosure walls and around the hut that was kept trimmed. Each enclosure contained water bottles, placed inside the hut, and feeding stations with lab chow (PicoLab Rodent Diet 20), placed outside the hut but connected to it by a covered wooden ramp. Our 2021 lab-housed mice (*n* = 61) were bred and raised at the same time in the same cages but rather maintained in standard laboratory husbandry conditions instead of being released. The protocols for the animal work (in 2017 and 2019) were approved by the NYU Langone Institutional Animal Care and Use Committee (IACUC) (#IA16-0087 and #IA16-00864). The protocols for mouse breeding in 2021 were approved by the NIAID IACUC; the protocols for releasing the laboratory mice into the outdoor enclosure facility and the rest of the work were approved by Princeton IACUC (#1982-17).

Variation in procedure among experimental years included the following: the duration of the rewilding period, the mouse genotypes used, the sexes of the mice used, the number of mice per enclosure, whether some mice were challenged with parasites, and the number of feeding stations per enclosure (see Supplementary Table S1). In 2017 and 2019, mice were rewilded for 7–8 weeks; in 2021 they were rewilded for 5 weeks. In 2017 and 2019, all the mice were C57BL/6 mice, with some mutants on the C57BL/6 background: *Atg16l1*^*T316A/+*^, *Atg16l1*^*T316A/T316A*^, and *Nod2*^−/−^ in 2017; *Dectin1*^+/−^ and *Dectin1*^−/−^, *Card9*^+/−^, and *Card9*^−/−^, and *Hand2*-NuTRAP in 2019. Because *Hand2*-NuTRAP mice are reporter mice, they are expected to be functionally immunologically identical to WT C57BL/6, and we have therefore classified them as such in our statistical analyses. By contrast, in 2021 we used three different highly inbred lab mouse strains: C57BL/6, 129S1, and PWK/PhJ. In 2017 we used both male and female mice, albeit separated into their own enclosures (two male-only enclosures and two female-only enclosures). In 2019 and 2021 we only used female mice. In 2017 we used ~30 mice per enclosure, in 2019 18–19 mice, and in 2021 ~15 mice per enclosure. Although these population densities are likely higher than those of wild mice [47], they are substantially lower than those experienced by lab mice in standard husbandry conditions, with as many as five mice in a cage <0.1 m^2^ [48]. In 2017 and 2019 mice were not given any experimental parasite challenges; in 2021 some mice were challenged with the gut helminth *Trichuris muris* to explore differing genetic susceptibilities to worm infections. Finally, in 2017 and 2019, two feeding stations were provided in each enclosure, whereas in 2021 only one feeding station was provided per enclosure. Weather also varied from year to year. There are thus some important differences between the years, but the effects of said differences on predictor and response variables are at least somewhat controlled for with a covariate of year, and specific factors like genotype, sex, and infection status are directly included in regression models to assess the importance of these variables.

### Laboratory methods

Our methods for sampling blood and MLNs from mice are first reported in [42–44]; we repeat them here for clarity. At the end of the experiment, the mice were trapped with baited Longworth and Havahart live traps, anesthetized via isofluorane inhalation, and sacrificed and had blood collected via cardiac puncture into heparin-coated tubes. In 2021, additional blood specifically for complete blood counts was collected prior to anesthetization and sacrifice via cheek bleeding with a 4mm Medipoint Golden Rod Lancet Blade (Medipoint Catalog #NC9922361) into heparin-coated tubes. In all cases, blood samples were immediately placed on ice. Further processing post-sacrifice included the removal of mesenteric lymph nodes and, in 2019, the spleen for assessment of lymphocyte populations and immune responses. Lab-housed mice were sacrificed at the same time and place with the same procedure, except only the use of traps.

We measured total immunoglobulin G and M (IgG and IgM) antibody concentrations with enzyme-linked immunosorbent assays (ELISA) performed on blood collected via cardiac punctures. We used kits from StemCell Tech for these assays: Mouse IgG ELISA Antibody Pair Kit (Catalog #01998) and Mouse IgM ELISA Antibody Pair (Catalog #01999). The basic procedures we used for the two kits are as follows. Plates were coated with 100 µl of capture antibody solution overnight at 4°C. They were then washed and blocked with 200 µl of incubation buffer, incubating the plates while covered for 2 h (IgG) or for 1 h (IgM) at 37°C. The plates were washed, and 100 µl of standard controls and samples were added to wells, with controls prepared according to the manufacturer’s instructions. Duplicates for each sample were always used for quality control. For both IgG and IgM we diluted plasma samples at 1:100 000; initial results indicated high concentrations, such that lower dilutions would not produce usable results. Once samples and controls were added, the plates were incubated while covered for 2 h at 37°C. The plates were then washed, and 100 µl of detection antibody solution was added to each well; the plates were then covered and incubated for 1 hour at 37°C. For IgG we proceeded immediately to the next step; for IgM, we then washed the plates and added 100 µl of streptavidin alkaline phosphatase (SA-ALP) solution to each well, incubating while covered for 1 h at 37°C. We washed the plates and added 100 µl of StemCell Tech p-nitrophenyl phosphate (pNPP) ELISA substrate (Catalog #01917) as a start solution. The plates were then incubated for 15 min, covered, at 37°C. After 15 min the absorbance of each well was read at 405 nm, with a second reading taken at 650 nm to control for any background interference. Concentrations for each sample were then calculated in R with the package “*drc,*” using the standards for a reference curve [49]; concentrations for a given sample were determined as the mean of the two duplicates. Samples with coefficients of variation between duplicates of greater than 10% were discarded and re-run on another plate.

Blood samples obtained via cheek bleeds in our 2021 experiment were analyzed for complete blood counts using an Element HT5 Veterinary Hematology Analyzer (Heska). After this analysis, the blood sampled from cheek bleeds and the blood sampled via cardiac punctures for each individual were mixed; in 2017 and 2019, this mixing was unnecessary because we did not collect any blood via cheek bleeds. The samples were spun for 10 min at 430 g to separate out the plasma. Plasma was stored at −80°C for analysis of cytokine concentrations. These concentrations of cytokines (here IL-6 and TNFα, since these were the only two cytokines that were assayed in both years) were measured using the commercially available murine Th cytokine LEGENDplex assay panel (Biolegend; Catalog #741044) following the manufacturer’s instructions.

The cellular fraction of the collected blood was resuspended with PBS, then separated via a gradient density separation method employing lymphocyte separation media (LSM from MP Biomedicals, LLC, Irvine, CA) following the manufacturer’s instructions. Peripheral blood mononuclear cells (PBMCs) were isolated, washed twice in phosphate-buffered saline (PBS), and prepared for flow cytometry. Single-cell suspensions were prepared from the MLNs by mashing the tissues through a 70 µm cell strainer and washing with RPMI medium, then washing the cells again with RPMI supplemented with 10% fetal calf serum (FCS). The single-cell suspensions prepared from PBMCs and MLN cells (kept separate) were washed twice with flow cytometry buffer (FACs Buffer) and PBS. The suspensions were incubated for 10 min at room temperature with Live/Dead Fixable Blue (Thermo Fisher) and Fc Block (clone KT1632, BD). Then mixtures of commercially available fluorescently conjugated antibodies were diluted in FACs buffer and 10% Brilliant Stain Buffer (BD) and added directly to the cell suspensions for a 30-min incubation period (for the precise antibodies used, see the relevant originating study). Cells were incubated in eBioscience Transcription Factor Fixation and permeabilization solution (Invitrogen) for 12–18 h at 4°C. Finally, the cell solutions were stained with fluorescently labeled antibodies against intracellular antigens diluted in Permeabilization Buffer (Invitrogen) for 1 h at 4°C. Flow cytometry data was collected on an Aurora spectral cytometer (Cytek); spectral unmixing was performed using single-strained controls using UltraComp eBeads (Invitrogen). Data were analyzed using the OMIQ software, with data cleaning and scaling performed with the FlowCut algorithm. To determine clustering, 10 000 live CD45+ cells were subsampled and re-clustered with the Joe’s Flow software (https://github.com/niaid/JoesFlow).

### Statistical methods

Our goal in this project was to assess the ability of ecoimmunology measures to predict more difficult-to-assay aspects of immune phenotype. This means that we were not *per se* attempting to identify causal relationships. Whether and how antibody expression is causally tied to abundance of CD4 T cells is subsidiary to whether the two are consistently correlated. A correlation can still help to predict immune parameters that cannot, for whatever reason, be measured directly. Assessment of causal relationships may be valuable for helping understand the conditions under which identified correlations will be present or hidden. Thus we perform two basic types of statistical analysis. First, we used models with just our simple, widely employable immune measure of interest as a predictor and an immune trait as a response variable, to explore whether these correlations are present (our “solo” models). Second, we used models with said widely employable immune measure and additional predictors to shed light on the relative influences of different factors on immune traits and the extent to which these widely employable immune predictors may only be informative in the presence of other pieces of information (our “complex” models).

All of our statistical analyses were conducted in R v4.1.2 [50]. We log_10_-transformed the widely employable immune measures prior to using them as predictors in regression models. Because all of our cell type abundances are relative abundances, rather than absolute abundances, they cannot be modeled as normally-distributed; instead, following the recommendation of [51], we use beta regressions, in which the response variable is on the interval (0,1). While it is hypothetically possible for these abundance variables to take on values of 0 or 1, we do not have any such cases in our dataset, and it is vanishingly unlikely in nature, such that it was reasonable for us to eschew zero-or-one-inflated beta (ZOIB) regressions. Our “complex” models for rewilded mice included genotype, year, mouse age, and length of time outside since initial release as additional predictors; for our studies of B-cell phenotype, we only used genotype as an additional predictor. In our complex models for lab-housed mice, we included only genotype and mouse age as additional predictors, because time outside is not applicable when the mice are lab-housed and because we only had mice from one experimental year (2021). Other possible predictors were sex and *T. muris* infection status; we discarded these two because models without them were preferred by model selection methods (here, Akaike information criterion). We always designated C57BL/6 as the reference value for the genotype factor in regression models, as it was the only genotype employed in all three experimental years and therefore could serve as a consistent point of contrast of genotypes while also allowing for more sensible incorporation of the variation by year. Because we used a substantial number of models with a wide range of predictors, we performed multiple-comparison corrections with *q*-values (which can be interpreted as the false discovery rate), calculated via the R package *q-value,* and with *q* < 0.05 as our threshold for statistical significance. We report *p*-values, *q*-values, estimated coefficients, and *R*^2^ values for each variable in each regression.

## Results

### Widely employable immune measures are influenced by genetics, experimental year

The data for our experiments come from three separate rewilding studies conducted in 2017, 2019, and 2021 (first reported in [42–45]). Experimental conditions differed among the experiments (Supplementary Table S1). The mouse strains used in each of these experiments differed, either only at single loci with documented effects on mouse immune phenotypes in the lab (2017 and 2019) or across the whole genome (2021). We used both male and female mice in 2017, but only females in 2019 and 2021; the duration of rewilding in 2021 was shorter (5 weeks vs. 7–8 weeks in 2017 and 2019). Population density—the number of mice per enclosure—also differed, as did the number of chow feeding stations, and in 2021 the mice were challenged with *Trichuris muris* two weeks after release. This heterogeneity in experimental conditions allows us to assess the relative influence of different factors on immune phenotypes and the extent to which the predictive capacities of simple immune measures are consistent across contexts.

**Figure 1:**
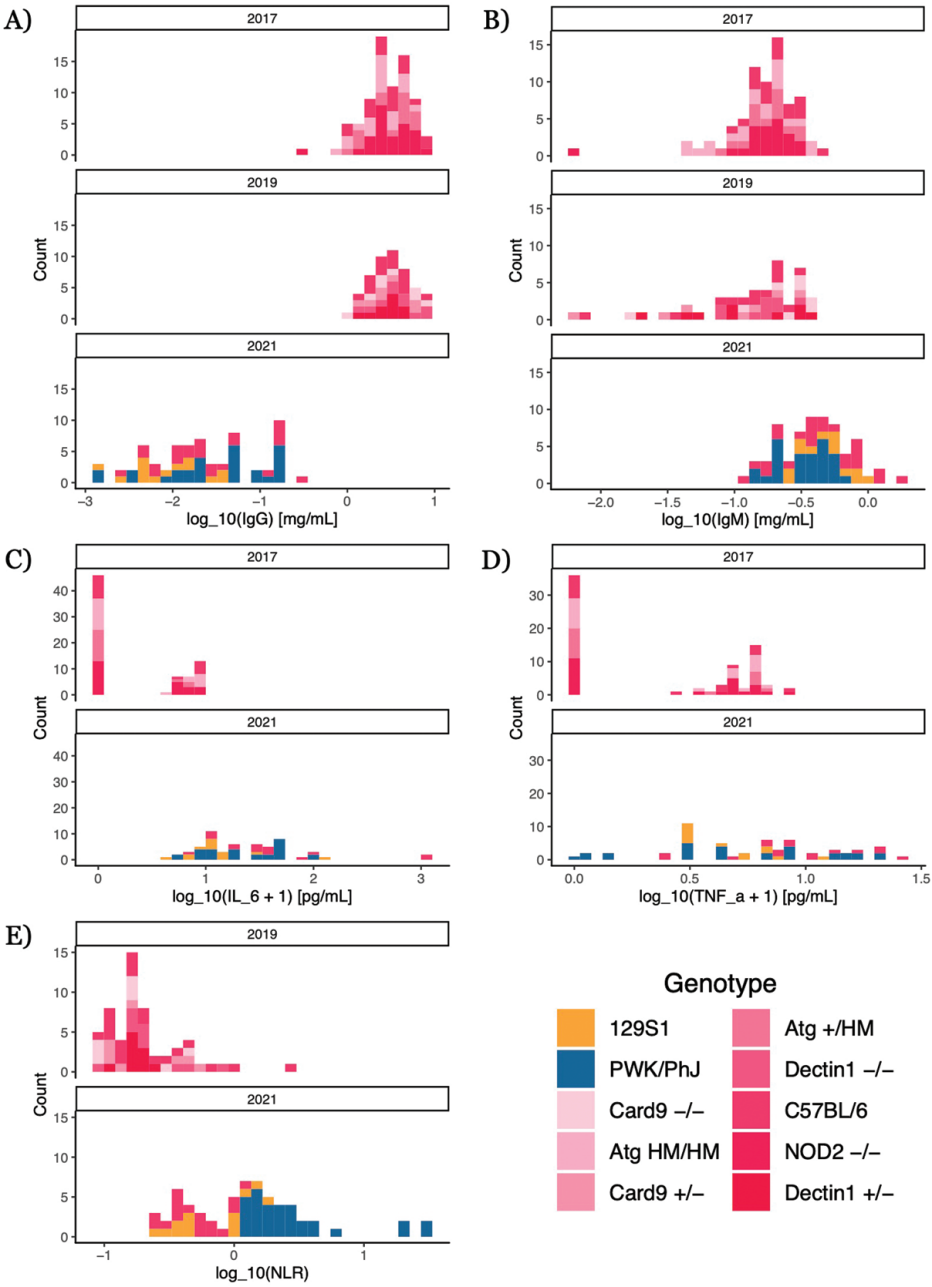
distributions of simple, widely employable immune quantities. Quantities were measured via ELISA (antibodies), bead-based assay (cytokines), and flow cytometry (neutrophil–lymphocyte ratio, NLR) (see Methods). Cytokine measures were not taken in 2019; NLR measures were not taken in 2017. Pink colors indicate mouse strains on a C57BL/6 background. (**A**) Total IgG concentration. (**B**) Total IgM concentration. (C)IL-6 concentration. (**D**) TNFα concentration. (**E**) NLR, broken down by genotype

We first analyzed variation in our five simple, widely employable immune measures: total IgG and IgM antibody concentrations in serum (Fig. 1A and B), IL–6 and TNFα concentrations in serum (Fig. 1C and D), and neutrophil–lymphocyte ratio (NLR) (all log_10_-transformed; Fig. 1E). These measures are ‘widely-employable’ because they can be performed relatively easily in samples collected from non-model organisms or in wild, settings—for example, NLR has been measured for a wide variety of mammal species [36]. After correcting for multiple comparisons using *q*-values, we found statistically significant effects (*q* < 0.05) from experimental year on the concentrations of some serum proteins: mice in 2021 had more IL–6 than mice in 2017 (2021 vs. 2017: 1.33 ± 0.243, *q* = 4.41 × 10^-6^, *p* = 2.36 × 10^-7^) and substantially less IgG (2021 vs. 2017: -1.99 ± 0.183, *q* = 1.04 × 10^-19^, *p* = 1.86 × 10^-21^) than mice in other years (Supplementary Fig. S1). Genotype (Supplementary Table S1, Materials and Methods) also influenced some of these simple immune measures, but statistically significant effects were generally confined to contrasts of different highly-inbred mouse strains; strains on the same C57BL/6 background, differing only at single loci, did not significantly differ from each other or from WT C57BL/6 for any of the widely-employable immune measures (complete numerical results are in Supplementary Appendix S1).

### A few of the widely employable measures correlate with particular lymphocyte populations

**Figure 2:**
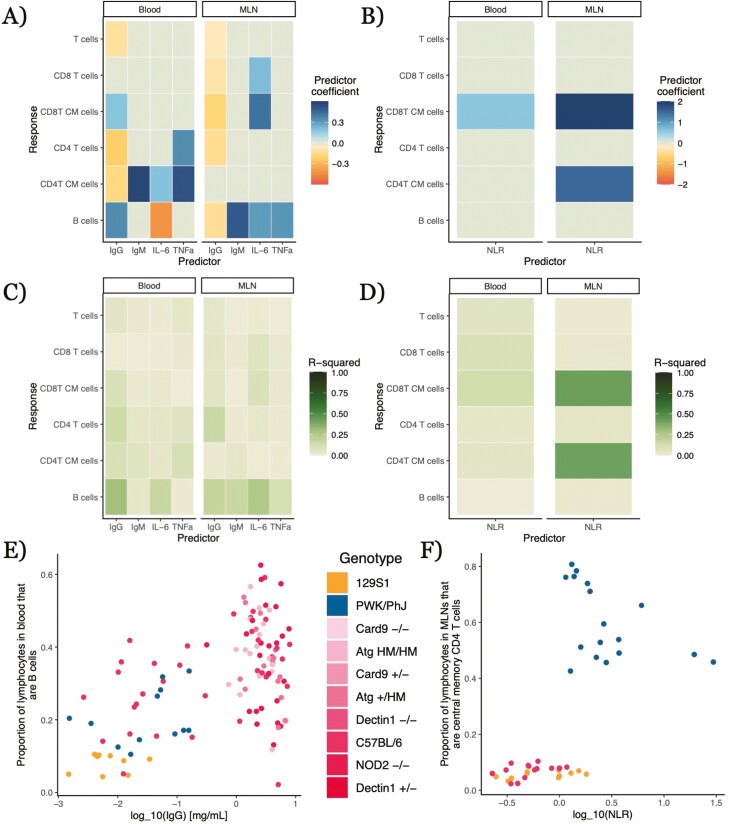
relationships between widely employable immune predictors and lymphocyte populations in solo models without other predictors. Target immune cell type relative abundance is modeled via beta regressions, with the widely-employable immune predictor log_10_-transformed. Color of a cell in A and B is the mean estimated coefficient value from the model; non-statistically significant relationships (*q* > 0.05) were given a coefficient value of 0 (the light gray-green color on the midpoint of our color scale). ‘CM’ in *y*-axis label stands for ‘central memory’. Complete numerical results in Supplementary Appendix S2. (**A**) Relationships between immune protein serum concentrations and lymphocyte types from solo models, broken down by tissue of lymphocyte type. (**B**) Relationships between neutrophil–lymphocyte ratio (NLR) and lymphocyte types from solo models, broken down by tissue of lymphocyte type. (**C**) *R*^2^ values for solo models of relationships between immune protein serum concentrations and lymphocyte types, broken down by tissue of lymphocyte type. (D) *R*^2^ values for solo models of relationships between NLR and lymphocyte types, broken down by tissue of lymphocyte type. (**E**) Total IgG, log_10_-transformed, and blood B-cell relative abundance. Pink colors indicate mouse strains on a C57BL/6 background. (**F**) NLR, log_10_-transformed, and mesenteric lymph node (MLN) central memory CD4 T-cell relative abundance

We explored how well our five simple immune measures could predict other immunological quantities of interest in statistical models without any covariates, like genetics or year of the experiment (henceforth referred to as ‘solo models’). The structure of these models is a basic beta regression to examine how well the log_10_ quantity of our widely employable immune measure predicts the proportional abundance of some lymphocyte type in a given tissue. These models test for predictive relationships that would particularly aid researchers working from samples without metadata (information on, e.g., environment or age) or available genetic information. We did find that simple immune measures have statistically significant correlations with a variety of factors in lymphocyte composition (*q* < 0.05 for 23 out of 60 analyses; Fig. 2A and B; complete numerical results are in Supplementary Appendices S2 and S3). However, these models generally had very low explanatory power, with *R*^2^ < 0.1 for 11 of the 23 statistically significant correlations and 48 of the 60 total analyses (Fig. 2C and D), indicating that these putative proxies capture only a small proportion of the variation in the quantities we wish to predict.

We did find a few strong relationships with modest *R*^2^ values for lymphocyte populations: IgG concentrations had a positive correlation with blood B-cell abundance (0.343 ± 0.0546, *q* = 1.76 × 10^−9^, *p* = 3.30 × 10^-10^, *R*^2^ = 0.296; Fig. 2E) and negative correlations with blood CD4 T-cell abundance (^−^0.218 ± 0.0448, *q* = 4.13 × 10^−6^, *p* = 1.17 × 10^−6^, *R*^2^ = 0.142) and MLN B-cell abundance (^−^0.138 ± 0.0259, *q* = 4.69 × 10^−7^, *p* = 1.14 × 10^−7^, *R*^2^ = 0.177). Neutrophil–lymphocyte ratio (NLR) had strong positive correlations with relative abundance of central memory CD8 (1.94 ± 0.303, *q* = 8.58 × 10^−10^, *p* = 1.56 × 10^−10^, *R*^2^ = 0.421) and central memory CD4 T cells in the MLNs (1.51 ± 0.269, *q* = 8.33 × 10^−8^, *p* = 1.88 × 10^−8^, *R*^2^ = 0.413; Fig. 2B and F). IL-6 concentration was negatively correlated with blood B-cell abundance (^−^0.427 ± 0.0931, *q* = 1.51 × 10^−5^, *p* = 4.51 × 10^−6^, *R*^2^ = 0.161) but positively correlated with MLN B-cell abundance (0.296 ± 0.0422, *q* = 1.45 × 10^−11^, *p* = 2.12 × 10^−12^, *R*^2^ = 0.250). These results all suggest there may be a few cases where widely employable immune measures offer predictive insight into otherwise unmeasured aspects of the immune phenotype and might therefore serve as useful proxies for aspects of cellular immune phenotype.

### Widely employable immune measures offer limited value as proxies even in the presence of other metadata

**Figure 3:**
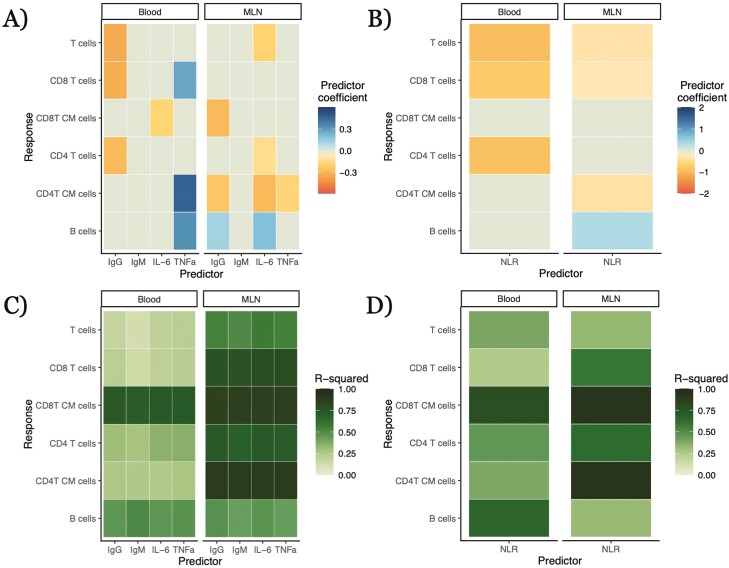
relationships between widely employable immune predictors and lymphocyte populations in complex models with other predictors. Other predictors in models were the year of the experiment, genotype (given as contrast with C57BL/6 WT), age of the mouse, and duration of time outdoors. Relative abundance of the target immune cell type is modeled via beta regressions, with the widely employable immune predictor log_10_-transformed. Color of a cell in A and B is the mean estimated coefficient value from the model; non-statistically significant relationships were given a coefficient value of 0 (the light gray-green color on the midpoint of our color scale). ‘CM’ in *y*-axis label stands for ‘central memory’. Complete numerical results in Supplementary Appendix S2. (**A**) Relationships between immune protein serum concentrations and lymphocyte types from complex models, broken down by tissue of lymphocyte type. (**B**) Relationships between neutrophil–lymphocyte ratio (NLR) and lymphocyte types from complex models, broken down by tissue of lymphocyte type. (**C**) *R*^2^ values for complex models of relationships between immune protein serum concentrations and lymphocyte types, broken down by tissue of lymphocyte type. (**D**) *R*^2^ values for complex models of relationships between NLR and lymphocyte types, broken down by tissue of lymphocyte type

**Figure 4:**
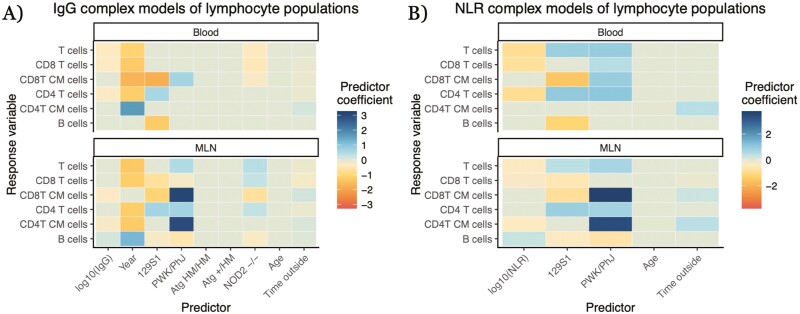
full results for complex models with other predictors of relationships between widely employable immune predictors and lymphocyte populations. Other predictors in models were a year of the experiment, genotype (given as contrast with C57BL/6 WT), age of the mouse, and duration of time outdoors. *Atg16l1*^HM/HM^, *Atg16l1*^HM/+^, and *Nod2*^−/−^ are all on a C57BL/6 background. Target immune cell type relative abundance is modeled via beta regressions, with the widely employable immune predictor log_10_-transformed. Color of a cell is the mean estimated coefficient value from the model; non-statistically significant relationships were given a coefficient value of 0 (the light gray-green color on the midpoint of our color scale). ‘CM’ in y-axis label stands for ‘central memory’. Complete numerical results in Supplementary Appendix S2. (**A**) Full model results including model-estimated coefficient values for all predictors in complex models of lymphocyte relative abundances with IgG as the immune molecule predictor. (**B**) Full model results from complex models of lymphocyte relative abundances with NLR as the immune molecule predictor.

We conducted further analyses incorporating metadata potentially relevant to the mouse immune phenotypes: age, genotype, and the length of time that the mouse had been housed outdoors, and the year in which the experiment was conducted (Figs. 3 and 4, Supplementary Fig. S2; complete numerical results are in Supplementary Appendices S2 and S3). These models we refer to as ‘complex models’, in contrast to the solo models, to reflect the increased number of predictors; they are also beta regression models predicting proportional abundance of lymphocyte types. A similar number of relationships between simple, widely employable immune measures and cellular immune phenotypes were statistically significant (22 out of 60), but only six relationships were statistically significant for both the complex (including both the proxy and other covariates) and the solo (only the proxy) models (Fig. 3A and B). Of those six relationships, five possessed the same sign in both the complex and solo models, while for one relationship—total IgG and B cells in the MLNs—the sign flipped between the two models. Several of the putative proxy measures correlating with lymphocyte phenotypes in the solo models, including total IgG as a positive correlate of B-cell frequencies in the blood (Fig. 2A), were no longer predictive, while new correlations came to light: e.g. total IgG was negatively associated with blood CD8 T-cell frequencies after including covariates (−0.435 ± 0.124; *q* = 0.0127; *p* = 7.70 × 10−^3^; Fig. 3A). For NLR, incorporation of covariates led to the intuitive negative correlation between NLR and T cells in the blood (Fig. 3B). The *R*^2^ value of each complex model with additional predictors was substantially higher than the *R*^2^ value of the corresponding solo model with the same widely employable immune predictor but without other metadata predictors (Fig. 3C and D). However, *R*^2^ values for the complex models of a given lymphocyte type were generally similar regardless of which immune protein was included as a predictor. These results again suggest that widely employable immunological measures are only partially predictive of lymphocyte phenotype even when additional metadata is known and can be used in an analysis.

One important aspect of our results is that coefficients for genetic differences and experimental year were frequently significantly greater in our models of lymphocyte populations than the coefficients for our putative proxy measures (Fig. 4, Supplementary Fig. S2). Highly inbred lab mouse strains had statistically significant differences (*q* < 0.05) from each other in immune cell abundances in the MLNs and blood in 50 out of 60 analyses. And even small genetic changes were sometimes associated with statistically significant differences in immune cell abundances. Knockouts of NOD2, which modulates recognition of bacteria [43], on a C57BL/6 background differed significantly in immune cell composition from wildtype (WT) C57BL/6 mice in 30 out of 48 analyses (Fig. 4, Supplementary Fig. S2). Such allele-specific differences could be similar or even greater in magnitude to those associated with the simple immune measures (accounting for the different scales of these predictors): e.g. CD8 T-cell abundance in the blood was negatively correlated with both IgG concentration (−0.329 ± 0.124, *q* = 0.0127, *p* = 7.70 × 10^−3^) and the NOD2^−/−^ genotype (^−^0.431 ± 0.155, *q* = 9.49 × 10^−3^, *p* = 5.44 × 10^−3^; Fig. 4A). Year was a statistically significant predictor in 34 out of 48 qualifying analyses (we did not have multiple years of data for our NLR dataset), often with large estimated effect coefficients—in the aforementioned model of IgG and blood CD8 T cells, the effect of experimental year (^−^1.26 ± 0.358, *q* = 9.68 × 10^−4^, *p* = 4.07 × 10^−4^) is approximately equal to the effect of a difference of four standard deviations of IgG concentration (Fig. 4A, Supplementary Fig. S2). Correlations between the proxy and target might even differ from year to year: a simple interaction model between IgG concentration and year for blood B cells, allowing for different IgG-B-cell relationships in different years, finds a negative correlation between IgG and blood B cells in the 2017 data (−0.502 ± 0.231, *p* = 0.0296) but a positive correlation in the 2021 data (IgG × 2021 effect: 0.980 ± 0.286, *p* = 6.16 × 10^−4^; Fig. 2E). Age generally had little identifiable predictive capability—although our mice span only a small subset of ages, being mostly early after maturation—but the duration of time outside (from the release of a mouse into its rewilding enclosure until its removal and sampling) often had very weak, statistically significant relationships with the quantities of interest, such as in the aforementioned IgG-blood CD8 T-cell model (^−^0.0740 ± 0.0250, *q* = 5.68 × 10^−3^, *p* = 3.09 × 10^−3^; Fig. 4A, Supplementary Fig. S2). Overall, we find that metadata offers more insights into how and why lymphocyte composition varies than do the widely employable immune measures, but the latter can supplement in some cases.

### Widely employable immune assays generally do not correlate with aspects of B-cell phenotype

Our results suggest that antibody concentrations, particularly IgG, and NLR hold the most promise as potential indicators of cellular immune phenotypes, having more correlations with lymphocyte composition in both solo and complex models than do serum cytokines. We next delved into specific subsets of B cells in lymphoid organs to explore whether concentrations of certain antibody isotypes can predict different aspects of an individual’s B-cell phenotype. We examined in our analyses different classes of B cells and samples from different tissues, including both the spleen and the MLNs; we did not have corresponding B-cell population data from the blood for these analyses. We found no statistically significant (*q* < 0.05) relationships between aspects of B-cell phenotype and abundance of either IgG or IgM in solo models (Supplementary Fig. S3A); in complex models with other predictors (here just genotype, as we only had data from one experiment in one year), we similarly found no statistically significant relationships between serum immune protein concentrations and B-cell phenotypes, with the closest being that between IgG and the relative abundance of IgM^+^ B cells in the MLNs (0.216 ± 0.123, *q* = 0.146, *p* = 0.0798; complete numerical results are in Supplementary Appendix S4). We did find statistically significant negative correlations between NLR and the abundance of B2 cells in the spleen in both the solo (−0.547 ± 0.157; *q* = 0.0141, *p* = 4.93 × 10^−4^; *R*^2^ = 0.225) and complex (^−^0.546 ± 0.143; *q* = 8.89 × 10^−3^; *p* = 1.34 × 10^−4^; *R*^2^ = 0.452) models. Genetic variation had statistically significant relationships with B-cell phenotype composition in a few of our models (Supplementary Fig. S3B–D). In our limited sample set (*n* = 41 for MLN B-cell phenotypes; *n* = 42 for spleen B-cell phenotypes), we do not have evidence that antibody concentrations are effective proxies for detailed aspects of an organism’s B-cell phenotype, but NLR may have some promise in this regard.

### Correlations identified in rewilded mice are not always present in laboratory-housed mice, and vice-versa

To further investigate the contingency of relationships between widely employable immune measures and lymphocyte concentrations, we took advantage of NLR and lymphocyte data from lab-housed mice in the 2021 experimental cohort, first reported in [41, 44]. We conducted similar analyses on this dataset with both solo and complex models (Supplementary Fig. S4; full numerical results in Supplementary Appendix S5). We found statistically significant correlations in 6 out of 12 solo models and 4 out of 12 complex models (Supplementary Fig. S4A and C). However, several of the correlations identified in our lab-housed sample were not present in the rewilded sample—for example, blood T cells negatively correlated with NLR in the solo model in lab-housed mice (−0.670 ± 0.360; *q* = 0.027; *p* = 0.063; Supplementary Fig. S4C), while there was no such statistically significant correlation in the rewilded mice. Conversely, some lymphocyte types that correlated with NLR in the rewilded mice, such as T and B cells, did not have correlations in the lab-housed mice (Supplementary Fig. S4C). These results suggest that correlations identified in one environmental context may not hold in other contexts, further illustrating the potential limits of widely employable immune measures as predictors.

### Relationships between lymphocyte abundances and widely employable immune measures rarely carry over across tissues

A given immune cell type exists in a variety of tissues in the body, often playing different roles in different contexts. Our dataset included abundances for several types of lymphocytes in both the blood and mesenteric lymph nodes. Correlations between a potential proxy protein measure and a lymphocyte type in one tissue were rarely duplicated for the same proxy and cell type in the other tissue analyzed (Fig. 3). For solo models, there were only three instances where corresponding relationships in both blood and MLNs possessed the same sign: the negative relationships between IgG and both CD4 T cell and overall T-cell abundances and between NLR and central memory CD8 T cells. In some cases the relationship was only statistically significant in one tissue—e.g. serum TNFα had a weak positive relationship with B-cell abundance in the MLNs (0.317 ± 0.0759, *q* = 0.0270, *p* = 2.96 × 10^−5^) but not in the blood (−0.111 ± 0.152, *q* = 0.109, *p* = 0.468). There were other cases where the relationships between the proxy and a cell type’s abundance in the two tissues were both statistically significant but inverted in sign, as in the relationships between IL-6 and B cells in the blood (negative) and MLNs (positive) or between IgG and blood B cells (positive) and MLN B cells (negative) (Fig. 3). For complex models, there were only two cell type–predictor pairs showing statistically significant relationships in the same direction across tissues: NLR and overall T and CD8 T cells (negative) (Fig. 3).

**Figure 5:**
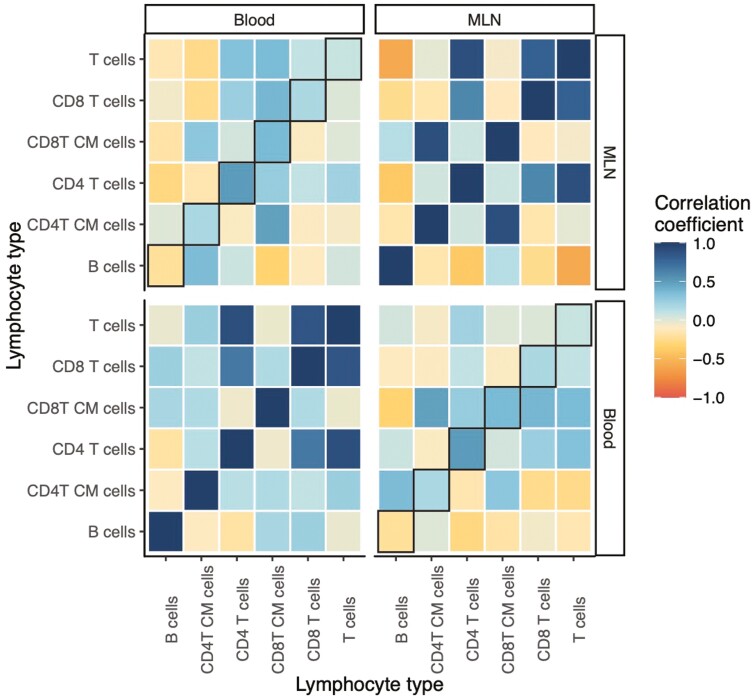
correlations among different classes of lymphocytes within and between tissues. Plot showing correlations between relative abundances of different lymphocyte types within and between tissues in rewilded mice. The correlation coefficient is calculated as Pearson’s *r*. Black box outlines indicate correlations between the same cell type in different tissues. ‘CM’ in y-axis label stands for ‘central memory’.

More generally, the abundance of a given lymphocyte type in one tissue did not always correlate well with the abundance of that lymphocyte type in another tissue (Fig. 5). We found a few strong relationships here: CD4 T-cell abundance was strongly positively correlated across the blood and MLNs (Pearson’s *r* = 0.501), while central memory CD8 T-cell abundance also positively correlated (*r* = 0.356). B-cell abundance in the blood was weakly negatively correlated with B-cell abundance in the MLNs (*r* = −0.215). But the three other cell types analyzed—T cells, CD8 T cells, and central memory CD4 T cells—all had weak correlations (|*r*| < 0.2). We did find strong positive correlations among the total, CD4, and CD8 T-cell abundances within each tissue, and B and T cells were negatively correlated, albeit only in the MLNs, highlighting the potential for inference of lymphocyte phenotype within a given tissue. However, the corresponding cross-tissue correlations were generally weaker. These results may go some way to explaining why there are few instances of consistent proxy-target correlations across tissues: the target cell types themselves rarely correlate well across tissues. And it was not the case that correlations are stronger for the broader cell type categories (e.g. T cells vs. central memory CD4 T cells); rather, which cell types correlate across tissues appears not to be predictable a priori.

## Discussion

Our analyses identified few cases where widely employable immune measures could function as proxies for lymphocyte composition. Immune quantities like lymphocyte composition are difficult or impossible to measure except when sampling model organisms in a laboratory setting, and the ability to indirectly estimate such quantities would offer researchers more insight into the immune phenotypes of wild organisms. However, while certain simple quantities, like serum concentrations of various proteins, can be measured in many taxa, our analyses here suggest that these easily-measured quantities have, at best, a weak relationship with variation in lymphocyte composition, and such relationships may only be recoverable with the collection of other data, such as sequencing of genetic markers. Thus the measurement of easily-accessible traits typically can only tell us with confidence those traits themselves, not what other, non-measured immune traits might be besides. In this respect, our results agree with those of [3], who found a lack of correlations between a number of immune traits of varying levels of detail in wild mice. Our results sound a note of caution for researchers attempting to describe immune phenotypes in non-laboratory conditions, but they also help delineate potential methods for unraveling the complexity of immune phenotypes.

We wanted to determine whether a readily measurable quantity can predict an unmeasurable one. One class of our analyses, the solo models, examined whether this can be achieved without incorporating other information about sampled individuals. Our results for these analyses are particularly relevant to researchers studying unmanaged populations that have not been sampled or tracked extensively. Any identified relationships that transcend other factors would allow researchers to characterize immune phenotype without needing these additional data (e.g. genetics or age) for each individual. However, ultimately we did not find many statistically significant relationships between our simple immune traits and harder-to-measure immune quantities. Even for statistically significant relationships, the coefficients of determination (*R*^2^) are almost uniformly small, suggesting low predictive utility. Our most promising results suggest that IgG and IL–6 concentrations in the serum do positively correlate with B-cell abundances in the blood and negatively with B-cell abundances in the MLNs. But in general our results suggest that serum antibody and cytokine concentrations mostly cannot effectively function on their own as proxies for other aspects of immune phenotype. Therefore we would caution researchers against inferring other aspects of immune phenotype and drawing conclusions about overall immune disposition or functionality based solely on serum concentrations of immune proteins.

Our complex models formed a second subset of our analyses, including as predictors not only the concentration of a serum immune protein but also other metadata, including an individual’s age, an individual’s genotype, and the year of the experiment. These analyses are more analogous to those employed on data collected from populations for which substantial metadata are available, either because they are directly managed populations or because they are wild populations subject to long-term monitoring. Our use of data from different experiments in multiple years here loosely approximates the collection of data across multiple sampling seasons for a given study population or multiple populations in different sites. Overall, model *R*^2^ values were higher for complex models than the corresponding solo models, suggesting these complex models may indeed offer more promise for identifying proxy relationships. However, genetic variation and year-on-year variation were almost invariably more associated with varied immune cell populations than were serum protein measures. We found that even small genetic differences, at a single locus, could produce substantial variation in the lymphocyte trait of interest. This poses an important inferential challenge for researchers. Genetic variation between individuals could be producing much more variation in target immune quantities than would be predicted from the proxy immune measure on its own. The different single-locus genetic variants we study here are at loci encoding proteins with important immunological roles, and they were chosen in the expectation of the sort of substantial immunological effects we observed [42, 43, 45]. So our results will likely not generalize across all types of genetic variation at all loci. However, the actual effects of genetic variants on the target trait will often be unknown because they require direct study. Thus determining the overall structure and causes of variation in immune phenotypes would potentially be impossible in a natural setting. Environmental heterogeneity factors, as analyzed here by the effect of year, the smaller but still statistically significant effects of duration of time outside, and the differences between our results in rewilded mice vs. lab mice, may further reduce our predictive ability in these cases. Researchers hoping to infer aspects of immune phenotype from proxies must still be cautious even if they have substantial metadata.

Two particular complicating phenomena further limit the assessment of these relationships: (i) relationships between a proxy and a target trait not carrying over across tissues and (ii) relationships between a proxy and a target trait differing in direction between the solo and complex models. The former phenomenon illustrates the limits of putative proxies—proxies may only be effective for a very small slice of an organism’s immune phenotype because there is a high contextual complexity to relationships between aspects of the immune system. Some of the widely employable immune measures did correlate with lymphocyte types in multiple tissues—we found in our solo models that greater serum IgG concentrations were associated with CD4 T cells being less common in both the blood and the MLNs. So perhaps IgG concentrations may inform researchers about CD4 T-cell disposition more systemically than, for example, IgM. But we identified few such relationships overall. This paucity reflects the complexity of the mechanisms that might link potential proxies and targets and the compartmentalization of the immune system. For example, although we found in our solo models that IgG concentration correlated positively with B-cell abundance in the blood after we included genotype as a predictor in our complex models, IgG concentration correlated negatively with B-cell abundance in the blood. These contrasting results may reflect different mechanisms of control of B cell and antibody abundance. Some genetic loci may affect IgG and blood B cells similarly, leading to positive correlations in models that do not condition on genetics. The negative correlations observed after controlling for genetic variation may reflect resource trade-offs on a given genetic background (antibody production is resource-intensive and could restrict the resources devoted to B-cell division). Layered on top of such trade-offs would be interactions between proxy traits, genetic variants, and/or environments, as suggested by our simple interaction analysis of the impact of the year on the estimated relationship between IgG and B cells and by the varying results we see between rewilded and lab-housed mice. And these will likely be quite different for different taxa. Our results suggest that researchers interested in basic levels of investment in humoral immunity can use antibody concentrations as a measure in the absence of other metadata. However, detecting trade-offs within humoral immune investment and understanding the evolution of investment in humoral immunity, as many researchers wish to do, requires an understanding of the genetic factors controlling antibody concentrations [18], B-cell phenotypes (e.g [43, 44].), and the relationships between them, illustrating the complexity of inferring detail in immune phenotypes solely from widely-employable immune measures.

One area of potential promise that we find is in the relationships between total serum IgG and the various lymphocyte types. IgG had a statistically significant relationship with the target lymphocyte type in 16 of 24 models—10 of the solo models and six of the complex models—spanning different lymphocyte types in different tissues. Solo models featuring IgG generally had higher *R*^2^ values than models with other potential proxy measures, although these values were still relatively low compared to the *R*^2^ values for corresponding complex models. Both T and B cells were predicted in multiple tissues, suggesting that further sampling, across a greater array of conditions and genetic backgrounds, may enable the identification with greater confidence of the relationships between IgG abundances and lymphocyte compositions across multiple tissues. Then IgG could be used as a weak indicator variable for at least a few aspects of immune phenotype. Although we do not find evidence of a relationship between total IgG (or IgM) concentrations and aspects of B-cell phenotype, we do only have small sample sizes, and abundances of different IgG subclasses may be more appropriate proxies here anyways. Accordingly, we do not want to reject the possibility of using IgG to infer B-cell phenotypes.

Neutrophil–lymphocyte ratio (NLR) also shows some promise as a proxy, albeit with many of the same issues described for antibodies. Some of the correlations we detected for NLR in solo models, like the positive correlation with central memory CD8 T cells, are likely products of the aforementioned effects of genotype on both the proxy and the target traits. However, it is particularly interesting that NLR negatively correlated with the relative abundance of B2 cells in the spleen. NLR is sometimes hypothesized to indicate, crudely, a relatively greater investment in innate immunity than adaptive immunity, given the respective functions and ontogenies of neutrophils and lymphocytes [5]. B2 cells, often known as follicular B cells, generally interact more with T cells and produce longer-lived immune memory than other B-cell subsets [52]. Accordingly, a negative correlation between the two could support NLR as an indicator of innate-adaptive balance. However, much as above, our small sample size, and the fact that we do not see the mirror-image correlation with relative abundance of the natural-antibody-producing B1 cells, should make us cautious about this result.

How should researchers approach the study of immune phenotypes in wild organisms, given these results? A key point that our results emphasize is that the focus, both immunologically and ecologically, should be on the quantity or quantities being directly measured. While our results suggest that serum protein concentrations offer limited insight into immune phenotypes, they are still of intrinsic interest to immunologists and disease ecologists. Antibody concentrations can be markers of resource investment patterns [53–55] and are correlated with various aspects of life history in several different species (e.g [10, 56–58].). They can be biomarkers for disease severity, such as the associations of high levels of serum IL–6 and high NLR with COVID-19 severity [59, 60]. Serum cytokine concentrations have even been tied to gregariousness in house mice [61]. But inferring other aspects of or broader trends in immune phenotype from these quantities requires substantial caution. If metadata are available, researchers can possibly estimate patterns of variation in unmeasurable immune traits with a higher degree of confidence, but our results suggest this variation will largely not be indicated by the serum protein concentrations, and the nature of said variation may be elusive. For example, if individuals vary genetically and in antibody concentration, genetic variation will likely better predict variation in immune cell phenotype—and cytokine responsiveness [43, 44] – than the concentration of antibodies. The concentration of antibodies may then help assess differences between genetically similar individuals, but not reliably between genetically-dissimilar individuals. Caution should be employed in drawing conclusions across steep environmental differences—our lab-housed vs. rewilded difference is an especially dramatic example.

Many of the potential pitfalls we outline here also apply to studies conducted in humans. The value of biomarkers and similar proxies is clear, particularly for diagnostic purposes. However, biomarker relationships identified from one cohort of individuals may not apply to another, as suggested by the interacting effect of year on the IgG-blood B-cell relationship that we document. The utility of a biomarker may only exist if certain other metadata is known, and it may not carry over to new contexts—new environments or new ranges of genetic variation—separate from the original cohort. Our data do not match the large sample sizes that can be used in studies identifying human biomarkers, but our results do hint that other aspects of individual variation may provide important context to understanding said biomarkers and when they are appropriate.

Our analysis is focused on intraspecific comparisons, but an additional challenge for researchers is whether any relationships identified here hold in other species. Although a relationship between a protein’s concentration and cell type may exist in one species, other species may have alternative networks of relationships between immune traits due to shifting genetic architectures, ecological contexts, and evolutionary processes [31, 62]. Thus researchers also must be careful when using serum protein concentrations as proxies in species that have seen little prior immunological study. In general, if the species of study is a model organism or one for which reagents for more detailed immunophenotyping exist, then researchers are better off directly assessing the trait or traits of interest.

Ultimately, our results remind us that there are likely not immune measures that can effectively summarize the overall state of the immune system. Simple, easily measurable immune traits have great value for researchers, both for what they tell us about the immune state and for what they might otherwise indicate about the disease ecology of the population. They may offer us some insight into relative variation in a few immune traits that we do not or cannot directly measure. However, they are not holistic descriptors of the immune system. Other methods—the development of new reagents, the use of controlled experimental conditions, etc. – are necessary for more comprehensively describing immune phenotype.

## Supplementary Material

kyae010_suppl_Supplementary_Materials

kyae010_suppl_Supplementary_Table_S1

kyae010_suppl_Supplementary_Table_S2

kyae010_suppl_Supplementary_Table_S3

kyae010_suppl_Supplementary_Table_S4

kyae010_suppl_Supplementary_Table_S5

## Data Availability

All data and code needed to replicate the analyses and figures and evaluate the conclusions of the paper are present in the paper and supplementary information and at https://github.com/aedownie/eco-immun-predict.

## Abbreviations

MHC: major histocompatibility complex
TLR: toll-like receptor
IgG: immunoglobulin G
IgM: immunoglobulin M
NLR: neutrophil-lymphocyte ratio
MLN: mesenteric lymph node
CD: cluster of differentiation
IL: interleukin
TNF: tumor necrosis factor
ELISA: enzyme-linked immunosorbent assay
PBMC: peripheral blood mononuclear cell
PBS: phosphate-buffered saline
