# Supplementary material for: Assessing immune phenotypes using simple proxy measures: promise and limitations
Source: Discov Immunol. 2024 Jun 28;3(1):kyae010. doi: 10.1093/discim/kyae010 (PMC11264049; doi:10.1093/discim/kyae010)
Supplement: kyae010_suppl_Supplementary_Materials [file kyae010_suppl_supplementary_materials.docx]

Supplementary Material

Figures S1–S4

Table S1

Appendices S1–S5 are in separate spreadsheet files

Assessing immune phenotypes using simple proxy measures: promise and limitations

Alexander E. Downie,^1*§^ Ramya S. Barre,^1,2^ Annie Robinson,^1^ Jennie Yang,^1^ Ying-Han Chen,^3,4,5^ Jian-Da Lin,^3,4,6,7^ Oyebola Oyesola,^8^ Frank Yeung,^3^ Ken Cadwell,^3,9,10,11^ P’ng Loke,^3,4,8^ Andrea L. Graham^1,12*^

^1^Department of Ecology and Evolutionary Biology, Princeton University; Princeton, NJ 08544, USA.

^2^Department of Microbiology, Immunology, and Molecular Genetics, University of Texas Health Sciences Center at San Antonio; San Antonio, TX 78229, USA.

^3^Kimmel Center for Biology and Medicine at the Skirball Institute, New York University Grossman School of Medicine; New York, NY 10016, USA.

^4^Department of Microbiology, New York University Grossman School of Medicine; New York, NY 10016, USA.

^5^Institute of Biomedical Sciences, Academia Sinica, Taipei City 115, Taiwan.

^6^Department of Biochemical Science and Technology, College of Life Science, National Taiwan University, Taipei City 10617, Taiwan.

^7^Center for Computational and Systems Biology, National Taiwan University, Taipei City 10617, Taiwan.

^8^Laboratory of Parasitic Diseases, National Institute for Allergy and Infectious Diseases, National Institutes of Health; Bethesda, MD 20892, USA.

^9^Division of Gastroenterology and Hepatology, Department of Medicine, University of Pennsylvania Perelman School of Medicine, Philadelphia, PA 19104, USA

^10^Department of Systems Pharmacology and Translational Therapeutics, University of Pennsylvania Perelman School of Medicine, Philadelphia, PA 19104, USA

^11^Department of Pathology and Laboratory Medicine, University of Pennsylvania Perelman School of Medicine, Philadelphia, PA 19104, USA

^12^Santa Fe Institute; Santa Fe, NM 87501, USA.

*Corresponding author: [alec_downie@eva.mpg.de](mailto:alec_downie@eva.mpg.de) (AED), [algraham@princeton.edu](mailto:algraham@princeton.edu) (ALG)

§Present address: Department of Primate Behavior and Evolution, Max Planck Institute for Evolutionary Anthropology, Leipzig, Germany

Figure S1: Factors affecting values of widely-employable immune predictors.

Immune protein serum concentrations modeled via Gaussian regressions after being log_10_-transformed. Predictors in model are year, genotype, age, and time outside. All genotype predictors are relative to C57BL/6 genotype, which was present in all years. *Atg16l1*^HM/HM^, *Atg16l1*^HM/+^, *Card9*^-/-^, *Card9*^+/-^, *Dectin1*^-/-^, *Dectin1*^+/-^, and *Nod2*^-/-^ are all on a C57BL/6 background. Year predictor is relative to 2017 experiment, except for NLR, where it is relative to 2019 experiment. Color of a cell is the mean estimated coefficient value from the model; non-statistically significant relationships were given a coefficient value of 0 (the light grey-green color on the midpoint of our color scale), while dark grey cells indicate that predictors was not included in the model of that response variable.

Figure S2: Full results for complex models with other predictors of relationships between widely-employable immune predictors and lymphocyte populations.

Other predictors were year of experiment, genotype (given as contrast with C57BL/6 WT), age of mouse, and duration of time outdoors. *Atg16l1*^HM/HM^, *Atg16l1*^HM/+^, and *Nod2^-^*^/-^ are all on a C57BL/6 background. Target immune cell type relative abundance is modeled via beta regressions, with the simple immune predictor log_10_-transformed. Color of a cell is the mean estimated coefficient value from the model; non-statistically significant relationships were given a coefficient value of 0 (the light grey-green color on the midpoint of our color scale). “CM” in y-axis label stands for “central memory.” A) Full model results from models of lymphocyte relative abundances with IgM as the immune molecule predictor. B) Full model results from models of lymphocyte relative abundances with IL–6 as the immune molecule predictor. C) Full model results from models of lymphocyte relative abundances with TNF𝛼 as the immune molecule predictor.

**Figure S3: Relationships between simple immune predictors and lymphocyte populations in models with genotype included.**

Genotype is given as contrast with C57BL/6 WT. *Card9*^-/-^, *Card9*^+/-^, *Dectin1*^-/-^, and *Dectin1*^+/-^ are all on a C57BL/6 background. Y-axis labels denote the tissue and B cell type of the response variable. Target immune cell type relative abundance is modeled via beta regressions, with the simple immune predictor log_10_-transformed. Color of a cell is the mean estimated coefficient value from the model; non-statistically significant relationships were given a coefficient value of 0 (the light grey-green color on the midpoint of our color scale). “CM” in y-axis label stands for “central memory.” A) Results from models of B cell phenotypes different immune molecule predictors and without genotype. B) Full model results from models of B cell phenotypes with NLR as the immune molecule predictor. C) Full model results from models of B cell phenotypes with IgG as the immune molecule predictor. D) Full model results from models of B cell phenotypes with IgM as the immune molecule predictor.

**Figure S4: Relationship between NLR and lymphocyte populations in laboratory mice, for both solo and complex models.**

Other predictors in complex models were genotype (given as contrast with C57BL/6 WT) and age of mouse. Relative abundance of the target immune cell type is modeled via beta regressions, with the widely-employable immune predictor log_10_-transformed. Color of a cell in A and C is the mean estimated coefficient value from the model; non-statistically significant relationships were given a coefficient value of 0 (the light grey-green color on the midpoint of our color scale). Color in B is the R^2^ of the statistical model. “CM” in y-axis label stands for “central memory.” A) Relationships between neutrophil-lymphocyte ratio (NLR) and lymphocyte types in solo models, broken down by tissue of lymphocyte type. B) R^2^ values for solo models of relationships between NLR and lymphocyte types, broken down by tissue of lymphocyte type. C) Full model results from complex models of lymphocyte relative abundances with NLR as the immune molecule predictor. D) R^2^ values for complex models of relationships between NLR and lymphocyte types, broken down by tissue of lymphocyte type.

Table S1: Differences among experimental years for data included in analyses.

Genotypes in 2021 are different highly-inbred laboratory mouse strains varying at large numbers of loci. Parasite challenge took place three weeks prior to trapout.

| Year of experiment | 2017 | 2019 | 2021 |
| --- | --- | --- | --- |
| Sex of mice | Female and male | Female | Female |
| Total number of mice released | 115 | 56 | 89 |
| Number of mice assayed for antibody concentration | 77 | 50 | 66 |
| Number of mice assayed for neutrophil-lymphocyte ratio | N/A | 56 | 76 |
| Population of each enclosure | ~29 | ~18 | ~15 |
| Duration of rewilding | 7–8 weeks | 8 weeks | 5 weeks |
| Parasite challenge | None | None | *Trichuris muris* (at two weeks post-release) |
| Genotypes | C57BL/6 and mutants on C57BL/6 background:  *Atg16l1^T316A/+^­*  *Atg16l1^T316A/T316A^*  *Nod2^-/-^* | C57BL/6 and mutants on C57BL/6 background:  *Dectin1^+/-^* and *^-/-^*  *Card9^+/-^* and *^-/-^*  *Hand2-*NuTRAP*­* | C57BL/6, 129S1/SvImJ,  PWK/PhJ |
| Feeding stations per enclosure | 2 | 2 | 1 |
